# Does Non-Food Cultivation of Cropland Increase Farmers’ Income?

**DOI:** 10.3390/ijerph19127329

**Published:** 2022-06-15

**Authors:** Wencai Yang, Caiyao Xu, Fanbin Kong

**Affiliations:** 1 College of Economics and Management, Zhejiang A&F University, Hangzhou 311300, China; yangwencai2022@126.com; 2 Research Academy for Rural Revitalization of Zhejiang Province, Zhejiang A&F University, Hangzhou 311300, China; 3 Institute of Ecological Civilization, Zhejiang A&F University, Hangzhou 311300, China

**Keywords:** non-food cultivation, cash crops, farmers’ income, Zhejiang, agricultural policy

## Abstract

The production of cash crops is often regarded as an effective way to increase farmers’ income. This study evaluates the impact of non-food cultivation of cropland on farmers’ income by using the least-squares (OLS) model in Zhejiang Province, eastern China. Farmers are further divided into different groups according to their income levels to analyze the different impacts of non-food cultivation on their household income. The result shows that non-food cultivation has a significant negative effect on farmers’ income, with a more pronounced effect on farmers with a relatively low income. Accordingly, the increase in the proportion of cash crops that are grown does not increase the income of farmers in Zhejiang; instead, this harms their income. Therefore, farmers in Zhejiang should not rely on the cultivation of cash crops for their prosperity but must focus on participating in non-farm employment to increase their household income.

## 1. Introduction

Due to the high added value that cash crops possess, they are widely regarded as a way to increase farmers’ income [1]. This is why so much farmers’ cropland, which was previously used for food crops, is now used for cash crops. In theory, the production of cash crops allows for households to increase their income and better meets their needs than food crops, and cash crops often have higher added value than food crops [2]. The Chinese government has also proposed and implemented the Cash Crop Poverty Alleviation (CCPA) project in low-income rural areas, which aims to improve agricultural performance and raise the income of farming households through improved crop varieties and changes in cropping structures [1]. In backward rural areas with a weak secondary and tertiary industry base, the cultivation of cash crops requires a lower investment and shorter production period than the development of industry and services, which is more in line with the local development requirements in a short period of time.

As stated in the previous literature, if a region’s economic base is poor, and the development of secondary and tertiary industries is backward and cannot provide sufficient jobs to farmers, the development of secondary and tertiary sectors takes a longer time. In the short term, it is difficult for farmers to earn income through employment in secondary and tertiary industries; therefore, it is reasonable for farmers in the region to increase their income by planting cash crops [1]. However, if, in economically developed areas, labor costs are high and the local area can provide sufficient non-farm jobs to allow for farmers to increase their total household income through non-farm income, then farmers choosing to plant cash crops may require them to spend more labor time in agricultural production and give up part of their non-farm income; this may lead to a decrease in total household income. Thus, the impact of growing cash crops on farm household income varies across regions. In addition, industry and services tend to contribute more rapidly to the increase in farm household income than farming, and the shrinkage of agriculture relative to industry and services can be observed in both developing and developed countries, with non-farm employment increasingly proving to be an effective way to help farm households increase their income and escape poverty [3].

The impact of cash crops’ cultivation on the income and welfare of farm households has been the focus of discussion among the community and scholars; however, the conclusions that are reached remain mixed, with some findings opposing each other. For instance, Masanjala found that tobacco cultivation had a positive and significant effect on household income [4]. Radchenko showed that cash crops’ cultivation increases harvest value and yield for farmers [5]. Arndt found that, although biofuels have replaced food crops to some extent, investment in biofuel production in Mozambique has contributed to growth and reductions in poverty [6]. Klasen found that, after the economic crisis in Indonesia, farmers were able to increase their income by 14% through cocoa cultivation, which played an important role in helping local farmers to emerge from the economic crisis [7]. The positive impact of cash crops’ cultivation on household income and welfare has been reported for cardamom production in Papua New Guinea, maize in Zambia, and potatoes in Rwanda [8,9].

However, it has also been concluded that cash crops’ cultivation by farmers has a negative effect on their household income and welfare. Tankari used Senegal as a study area and found that, due to the high opportunity costs involved in cash crops’ production, cash crops’ cultivation has a negative effect on household welfare [10]. Anderman found a significant negative relationship between household welfare and the intensity of cash crops in Ghana [11]. Li divided the impact of cash crops cultivation on farm household income into two components: farm income and off-farm income, taking low-income regions in China as the study area. Cash crops’ cultivation was found to require that farming households make adjustments in labor allocation. As more time is devoted to agricultural production, off-farm labor decreases; this leads to an increase in farm income and a decrease in off-farm income for farm households [1]. Another issue related to cash cropping is the risk associated with this activity, including harvest failures (due to pests, diseases, and drought), price slumps, loss of market access, and a decline in income [12]; this makes the returns from cash crops’ cultivation more uncertain. Although some of the literature studied the impact of cash crops on farm household income, these studies mostly used micro-survey data to analyze the impact of the scale of cash crops’ cultivation on their household income, with less research on changes in cropping structure (based on the given cropland, a shift in cropping structure from food crops to cash crops) and the impact of this change on their household income returns.

In recent years, non-food cultivation (in this paper, food crops are defined as a collective term for cereal crops, potato crops, and legumes) of cropland has become a common phenomenon in Zhejiang; the area sown for food crops has decreased from 64.7% of the total sown area in 2000 to 49.3% in 2020. Non-food cultivation poses a threat to food security. The government has taken note of the far-reaching implications of non-food cultivation on food security, and policies have been enacted to curb this trend: on 22 July 2021, the general office of Zhejiang issued the “general office of Zhejiang provincial people’s government on resolutely stopping the ‘non-agriculturalization’ of cropland to prevent the non-food of the cropland to stabilize the development of food production”. Cash crop cultivation often needs more labor relative to food crops. It requires farmers to develop a trade-off between the time used for farm work and off-farm work, which means that non-food cultivation impacts farmers’ farm income and off-farm income.

Given the important impact of non-food cultivation on farm household incomes, and the relative vacancy in the field of analysis of the impact of farmers’ changes in planting structure on their income, there is a need to evaluate the effects of non-food cultivation on farmers’ household income and understand whether non-food cultivation increases farmers’ income. Therefore, this study takes Zhejiang as the study area and attempts to extend the existing studies by analyzing the impact of non-food cultivation on household income. Further, according to farmers’ disposable income levels, we divided farmers into five groups: low-income (20% of farmers with the lowest income level), sub-low-income (20% of farmers with the next lowest income level), middle-income (20% of farmers with the medium income level), sub-high-income (20% of farmers with the next highest income level), and upper-income (20% of farmers with the highest income level). We use this to discuss separately the impact of non-food cultivation on farmers’ household income, and analyze whether there are differences in the impact of non-food cultivation according to farmers’ different income levels. We used the least-squares (OLS) model to analyze the impact of non-food cultivation on farmers’ household income. To conduct a robustness check and comparison, we also presented the results, as estimated by the second-order least square (IV-2SLS), and tested the results, by changing the explanatory variables and adding relevant control variables to enhance the credibility of the results, to analyze the possible impact on farm household income that is obtained by following the curb-non-food-cultivation policy. This has important implications for the design and implementation of effective agricultural policies, and provides a reference for the use of green finances to help develop the green food industry.

## 2. Study Area Overview and Theoretical Framework

### 2.1. Study Area Overview

**Figure 1:**
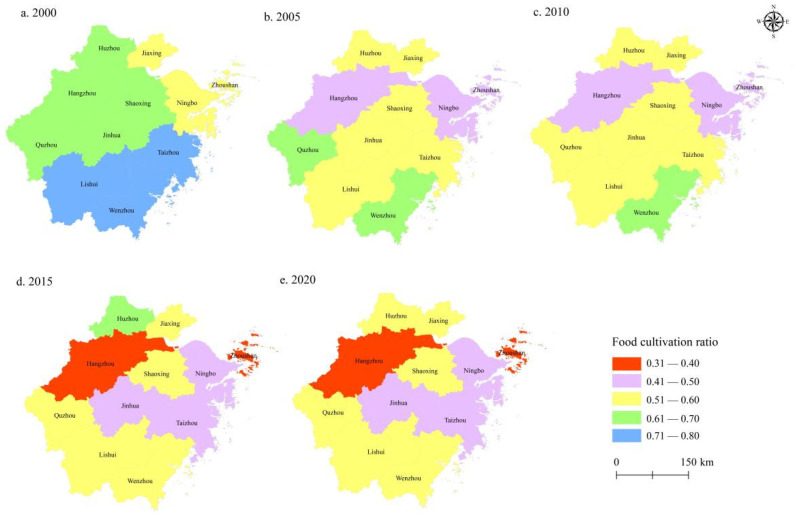
Trend of food planting ratio of cropland in Zhejiang province from 2000 to 2020.

Zhejiang is located on the eastern coast of China and is a major food marketing area in China. In recent years, the non-food cultivation of cropland in Zhejiang has become increasingly serious. As shown in Figure 1, all prefecture-level cities in Zhejiang have experienced varying degrees of non-food cultivation of cropland. The proportion of food cultivation in Hangzhou, Ningbo, Zhoushan, Jinhua, and Taizhou all dropped to less than 50%. The two cities with the most serious non-food cultivation, Hangzhou and Zhoushan, have only 36.6% and 33.8% of their cropland used for food cultivation. However, the increasing proportion of cash crops grown has not led to an obvious increase in business income.

**Figure 2:**
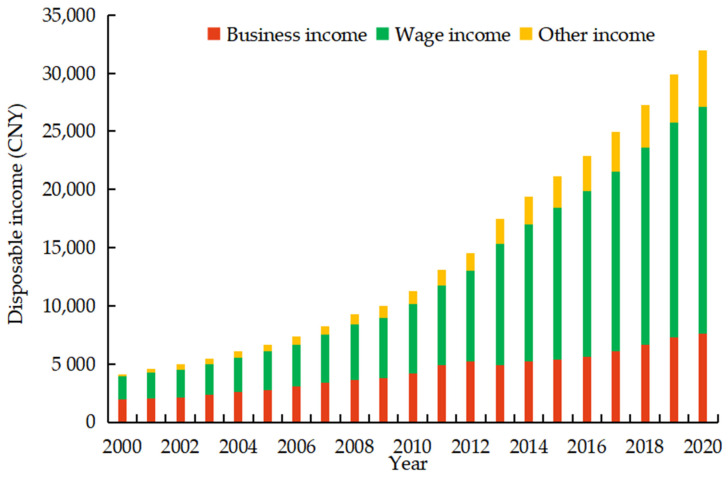
The composition of disposable income of farm households in Zhejiang province from 2000 to 2020.

As shown in Figure 2, the proportion of the disposable income of rural residents in Zhejiang has been expanding in terms of wage income and shrinking in terms of business income. The contribution of business income to the growth in farm household income is decreasing; this proves that secondary and tertiary industries are an effective way for farmers to escape poverty and achieve income growth, as previously suggested by scholars. The fluctuating, slow growth in total business income shows that farmers in Zhejiang do not earn more business income from non-food cultivation. 

### 2.2. Theoretical Framework

When farmers choose to plant cash crops or food crops, a major concern is how to maximize their income. For example, in Pakistan, the majority of farmers are willing to grow genetically modified cash crops, while only a few farmers are willing to grow genetically modified food crops. An important reason for this is that they believe that the cultivation of cash crops can increase their income [13]. Farmers in Mozambique find that growing biofuels can help to lift them out of poverty, meaning that biofuels are gradually replacing food crops in the region [6]. The positive contributions of cardamom production in Papua New Guinea and potatoes in Rwanda to household income all promote the local planting of these cash crops [8,9]. However, the cultivation of cash crops requires farmers to invest more labor time and capital; this requires farmers to give up a certain amount of their non-agricultural income. Therefore, the cultivation of cash crops does not have a definite positive impact on the income of local farmers, as the mechanism of its impact on farmers’ income should be considered from various perspectives.

**Figure 3:**
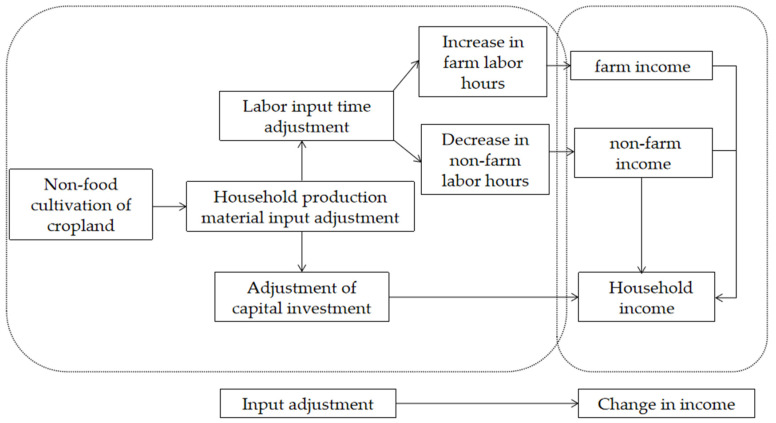
The relationship between non-food cultivation and household income.

A simple framework of these possible mechanisms is illustrated in Figure 3. Cash crops’ cultivation may require rural households to re-allocate the time that is used for off-farm activities. For example, allocating more time to cash crops’ cultivation would reduce the time used for off-farm work, and this would reduce off-farm income. Therefore, cash crops’ cultivation may not only have an impact on farm income but could also affect off-farm income, and off-farm income has an important role in raising the total income of farm households. When farmers shift from growing food crops to cash crops, both more labor and more capital investment are required. In addition, farmers need to take increased business risks. All these factors affect farmers’ total household income. Whether increasing the proportion of cash crops has a positive effect on farmers’ household income is an important issue.

## 3. Data and Method

### 3.1. Data

This study takes 2000–2020 as the research period, and the data were obtained from the Statistical Yearbook of Zhejiang province, the Statistical Yearbook of each prefecture-level city in Zhejiang Province, the China Statistical Yearbook, and some relevant data obtained through consultation with relevant departments. The missing data for individual years were calculated by applying the linear fitting method to the data of the years before and after to obtain estimated values. Detailed descriptions of the variables and statistical indicators are shown in Table 1.

### 3.2. Variable Selection

1. Explained variables: Disposable income (take logarithm). Disposable income is considered the most important determinant of consumer spending, and thus is often used to measure changes in a household’s standard of living. According to the source of income, disposable income consists of four items: wage income, business income, property income, and transfer income.
2. Core explanatory variables: Percentage of cash crops that are sown. The proportion of cash crops sown reflects how farmers choose to allocate their cropland resources given limited cropland resources, which is more in line with the research needs of this paper. Therefore, we choose to use the proportion of cash crops sown instead of the total cash crop area as the core explanatory variable.
3. Control variables. The control variables in this study refer to previous studies [14,15,16]. Control variables were introduced from economic and production dimensions. Among the economic factors are GDP and urbanization; among the production factors are mechanization level, education level, primary industry employment, and road mileage. The selection of control variables was based on the following:
  (1) GDP (take logarithm). The total GDP reflects the economic base and development of a region; the better the economic conditions of a region, the better the local industry and infrastructure will be, which can provide more off-farm jobs for local farmers and provide financial and technical self-sufficiency for agricultural production, thus affecting farmers’ income. At the same time, with the development of the economic level, people’s consumption of agricultural products also changes, as the demand for green agricultural products increases, which influences farmers’ planting choices.
  (2) Mechanization. Considering that the total mechanized power is influenced by the total area of crops sown, it is not simple to evaluate the increase (decrease) in the mechanized level of agricultural production mechanization by the increase (decrease) in the total mechanized power. Therefore, this paper uses the mechanized power per acre to measure the mechanization level. Improvements in the mechanization level can reduce labor input in the process of agricultural production, saving labor costs and allowing farmers to devote more time to other production activities.
  (3) Education level. The higher the number of years of education, the more knowledge and skills farmers have, the more willing they are to accept new things, and the more job opportunities they have. This makes educated farming households more inclined to non-farm employment, and the reduction in agricultural labor may lead to a tendency for farming households to grow labor-saving food crops, which also has a certain impact on their income.
  (4) Primary industry employment (take logarithm). The number of people employed in the primary sector reflects the number of rural laborers engaged in agricultural production. Theoretically, when more people are engaged in the primary sector, farmers tend to grow more labor-intensive cash crops, have a higher farm income and receive a lower non-farm income.
  (5) Urbanization. Population urbanization and land urbanization are important signs of urbanization. Population urbanization implies an increase in employment opportunities and an expansion of income sources, as well as increased access to secondary and tertiary income. This also leads to a reduction in agricultural labor, and labor-saving food crops become a farming option, which changes the income structure and affects the income of farm households. Therefore, this position uses population urbanization as an indicator of the level of urbanization.
  (6) Road mileage (take logarithm). Road mileage reflects the infrastructure development of a region. The longer the road mileage, the higher the accessibility between regions, which makes it easier for farmers to go out to work. Improved transport conditions have made it easier and faster to transport crops, providing better conditions for cash crops, which have a shorter shelf life. This affects farmers’ planting choices and income.

### 3.3. Method

The explanatory variable “disposable income” is a continuous variable; thus, this study referenced WU’s approach, and used least-squares (OLS) regression analysis to determine how non-food cultivation of cropland influences farmers’ disposable income [17]. The equation for factors that influence farmers’ disposable income is formulated as follows:

(1) $$Y_{\mathrm{it}}=\beta_{0}+\beta_{1}U_{t}+\beta_{2}X_{\mathrm{it}}+\varepsilon_{i}$$

where $Y_{\mathrm{it}}$ is the different disposable income levels of farm households; t means different years;$\;U_{t}$ is used for the non-food cultivation of cropland in different years;$\;X_{\mathrm{it}}$ is used for the control variables; $\beta_{0}$, $\beta_{1}$, $\beta_{2}$, are the parameters to be estimated; $\varepsilon_{i}$ is the error.

Considering the possibility of endogenous problems, this article also used the estimation results of the 2SLS model to examine the robustness of the estimation results. This article selected the proportion of national cash crop sown area as an instrumental variable; there is no logical link between the national proportion of cash crops sown and the disposable income of farm households in Zhejiang, meeting the exogenous requirements.

In this paper, the first stage was estimated by the following equation:

(2) $$U_{t}=\omega_{0}+\omega_{1}{\mathrm{IN}}_{t}+\omega_{2}X_{\mathrm{it}}+\zeta_{i}$$

where $U_{t}$ refers to the non-food cultivation of cropland in different years; ${\mathrm{IN}}_{t}$ to the instrumental variable “the proportion of national cash crops sown area”; $X_{\mathrm{it}}$ has the same meaning as Equation (1); $\zeta_{i}$ is the error; $\omega_{1}$ is the instrumental variable coefficient. A weak instrumental variable can be estimated by whether its F-statistic is greater than the empirical value of 10.

On the basis of (2), the second stage least squares model is constructed:

(3) $$Y_{\mathrm{it}}=\eta_{0}+\eta_{1}U_{t}+\eta_{2}X_{\mathrm{it}}+\delta_{i}$$

where $Y_{\mathrm{it}}$ is the different disposable income levels of farm households;$\;\delta_{i}$ is the error; the rest of the variables are the same as in Equation (1).

## 4. Results and Discussion

Table 2 presents the impact of non-food cultivation of cropland on the non-farm and farm incomes of farm households. Our estimates show that the coefficient of the percentage of cash crops sown has a negative and statistically significant impact on farmers’ off-farm income, indicating that cash crops’ cultivation reduces the off-farm income of farm households. Moreover, from the analysis in Table 2, it is concluded that the percentage of cash crops does not yield the desired significant positive impact on farm income. Thus, it is reasonable to suspect that such non-food cultivation in Zhejiang does not increase the total income of farm households; instead, there is a negative impact on the total income of farm households. To test our ideas, in the next section, we analyze the impact of non-food cultivation on the total income of all farmers and of farmers in different income brackets.

Table 3 presents the results regarding the impact of the percentage of cash crops sown on household income. The results show that the coefficient of the percentage of cash crops sown in the model is negative and statistically significant for farmers’ household income, suggesting that an increase in the proportion of cash crops decreases household income. In general, food crops have a higher rate of mechanization than cash crops and can easily participate in the division of labor in agriculture, with typical labor-saving characteristics. As cash crops are a labor-intensive crop, they require farmers to allocate more of their off-farm labor time to agricultural labor. Moreover, the predominantly hilly terrain of Zhejiang makes agricultural work more difficult, meaning that farmers need to invest more time; in this case, farmers need to give up more of their off-farm labor time, which leads to a lower off-farm income for farm households [1]. Zhejiang, a developed province in eastern China, has higher labor wages, increasing farmers’ non-farm income, while the increase in farm income from growing cash crops is not obvious. Cash crops’ cultivation, as was found in many other studies [18,19,20], can make farmer’s income susceptible to crop failure, price fluctuation, inefficient market institutions, and exploitation by buyers. All of these influencing factors have resulted in non-food cultivation having a negative income on farmers’ income in Zhejiang.

From the regression results, we can also find that the percentage of cash crops has a greater negative impact on the low-income farming households, while it has no significant effect on the income of upper-income households. Farmers with lower incomes have limited access to market information and are unable to make timely adjustments to changes in market demand. This means that they are more easily influenced by fluctuations in agricultural prices. Additionally, low-income farmers have a lower level of agricultural mechanization than high-income farmers; this requires them to devote more time to agricultural labor and further reduces their non-farm income, meaning that non-food cultivation of cropland has a greater negative impact on low-income farmers’ households. Upper-income farmers are less or not engaged in agricultural work, and mainly earn their income from secondary and tertiary sectors. They are also not required to establish a trade-off between the time being allocated to farm work and the time being allocated to off-farm work, so an increase in the proportion of cash crops being grown does not have much impact on them.

For other factors that affect household income, our results were generally in line with theoretical expectations, and broadly consistent with the findings of several previous studies [14,15,16]. Specifically, the coefficient of GDP is positive and statistically significant, suggesting that the development of the regional economy can lead to an increase in farmers’ income. The overall positive economic development of the region can provide sufficient non-farm jobs for local farmers, improve rural infrastructure, and increase farmers’ security, contributing to an increase in household income. Furthermore, the regional economy was also found to have a high positive effect on higher-income-level farmers, while the effect on low-income farmers was not significant. This suggests that the dividends of regional economic development are well-placed to be enjoyed by high-income farmers but are rarely harvested by low-income farmers. Mechanization levels appear to have a positive and statistically significant coefficient. This finding suggests that the level of mechanization in agricultural labor can increase productivity and reduce the time spent on agricultural labor, allowing farmers to devote more time to off-farm production and increase their household income. As previously stated, upper-income farmers have little or no involvement in labor productivity in the agricultural industry, so the impact of agricultural mechanization on their income is not significant. The variable of education has a positive and statistically significant impact on household income, indicating that farmers who can access better education can obtain a higher household income. Better education can help farmers to acquire new knowledge and technology, make them more competitive, allow them to obtain better-paid positions and increase farm household income. The lack of significant benefits derived from education being experienced by low-income farmers may be due to the low priority given to education by low-income farmers, resulting in them not being well-placed to achieve significant increases in income through education.

## 5. Robust Test

### 5.1. Instrumental Variable Estimation

Endogenous problems may be faced when analyzing the impact effects of non-food cultivation on farmers’ household income, leading to inconsistent or biased parameter estimation results. There are two main reasons for this endogenous problem. On the one hand, there may be a two-way causal relationship between the non-food cultivation of cropland and farm household income. In general, the lower the household income, the fewer productive resources a farm household has, and the less competitive the labor force itself. They may not be able to obtain suitable non-farm jobs, leading them to farm cash crops for income. Conversely, households with higher income levels tend to be able to find positions with relatively higher salary levels and obtain a higher source of income than through cash crop farming, making them less likely to engage in cash crop farming. However, the baseline model may omit variables. Farmers make their own decisions about whether to grow cash crops, and the factors that influence their decisions are both observable and unobservable, which leads to selectivity bias and inconsistency in our analysis [5,21].

To overcome the possible endogenous problems shown above, instrumental variables for the percentage of cash crops sown were originally searched, and the effect of the percentage of cash crops’ sown on the income level of farm households was measured using the second-order least square (IV-2SLS). This paper used the proportion of national cash crops’ sown area as an instrumental variable. Table 4 reports the estimated results. From the results of the instrumental variables test, the F-value of the first-stage weak instrumental variables test are shown to be greater than the usual criterion (F-value of 10); therefore, the instrumental variables in this paper are acceptable. Focusing on the endogenous factor, the proportion of cash crops has a greater negative impact on lower-income farming households, and there is no significant effect on the income of upper-income households. Looking at farmers in each income bracket, the regression results remain consistent with the previous baseline model estimates in the direction of the coefficients, and there is a significant increase in the magnitude of the coefficients, which generally does not affect the findings of the baseline model. To ensure the reliability of the findings, further robustness tests were conducted using other methods.

### 5.2. Changing the Explanatory Variables

Disposable income not only includes business income and wage income, but also property income and transfer income. Therefore, using the disposable income levels of farm households to measure the impact of non-food cultivation on farmers’ household income may also be biased, affecting the reliability of the estimation results. To resolve this problem, this paper references Cuong’s (2009) approach, which uses the living consumption expenditure of rural residents as the explanatory variable to measure the impact of non-food cultivation on farmers’ household income (in general, the higher the income level of farm households, the higher their total living consumption expenditure) [22]. The estimated results are shown in Table 5. The estimated results show that an increase in the percentage of cash crops’ sown significantly reduces the subsistence consumption expenditure of farmers, especially for farmers with relatively low incomes, while the effect on upper-income farmers is not significant. For both the Zhejiang sample and the sample of farm households in different income classes, the estimation results are in perfect agreement with the baseline model estimates in terms of direction. This means that, at least in terms of the direction of action, the use of indicators similar to disposable income still leads to consistent findings; thus, the estimates of the benchmark model are generally robust and reliable.

### 5.3. Additional Variables Method

The problem of omitting variables arises when we study the impact of non-food cultivation of cropland on farmers’ household income. The problem of omitted variables is unlikely to be completely solved. To minimize the impact of omitted variables on the results of this paper, based on previous research [11,23,24], this study adds two control variables that were mentioned in previous papers: household size and the proportion of rural females. Table 6 reports the results of the estimation of the relevant parameters. The results of the parameter estimate for both the Zhejiang sample and the sample of farmers at all income levels are consistent with the baseline model in terms of direction, and only slightly differ in terms of the magnitude of the coefficients. Accordingly, this paper concludes that the effect of non-food cultivation on farm household income, when analyzed using the benchmark model, is robust to a certain extent and is not the result of chance. When generated by a particular estimation method, the results show a certain degree of confidence.

## 6. Discussion

As a developed Chinese province with a predominantly hilly terrain, Zhejiang’s finely fragmented cropland and high labor costs mean that cash crops’ cultivation has a high opportunity for increased costs. This analysis aimed to further our understanding of the impact of non-food cultivation of cropland on farmers’ household income, which is important in a developed province with high levels of non-food cultivation. A key finding is the negative impact of non-food cultivation on the household income of farmers in Zhejiang. This result is in line with other findings showing the negative effects of cash cropping in post-war Mozambique and Malawi [11,25], which also support the view that cash crops’ cultivation may increase farmers’ poverty [26]; however, these findings were contrary to previous conclusions that the cultivation of cash crops can increase farmers’ income [4,5,6]. This is also in line with the previously mentioned findings that the impact of growing cash crops on farm household income varies across regions. Therefore, the impact that the planting of cash crops has on farmer’s income is worthy of further in-depth study.

Furthermore, the study revealed a heterogeneous effect of cash cropping on farmers’ household income in Zhejiang. The negative impact of non-food cultivation was more severe for farmers with a relatively low income, while there was no significant impact on upper-income farmers. This finding is in line with the conclusion drawn from the study of Senegal [10]. Generally, farmers with lower incomes have limited information and are slower to respond to changes in market demand. In addition, a good selling price for their production is not guaranteed, and farmers with a relatively low income mostly practice fine-grained farming, with low levels of mechanization, which requires more hours of farm labor. This reduces their off-farm income, which increases the negative impact of non-food cultivation.

It is important to emphasize that the results of this study are not intended to refute the benefits of the commercialization of agriculture, but rather aim to call for the restructuring of production systems, and call on farmers to choose labor-saving food crops on limited cropland as much as possible. The government and related departments should take a multi-faceted approach to promoting changes in farming structures among farmers, promoting the development of the green food industry, and providing more off-farm jobs for farmers, as well as promoting labor-saving food crops by reducing the number of people working in agriculture.

The government should take measures to promote the establishment of food branding in Zhejiang and improve market competitiveness, build a pilot project for the development of smart agriculture, increase the research and application of drones, sprayers, and other equipment, and build a smart food production workshop with the help of the Internet of Things, big data, and the Cloud. The company is also building a smart food production workshop to achieve intelligent processing, control, and decision making, improve the food traceability system, reduce the rate of broken food, and increase the rate of refined food. At the same time, support for the leading industry should be strengthened, the brand image of food enterprises should be shaped through professional management, product grading, and quality control should be carried out, and credit support and financial product innovation should be used to promote the branding of food enterprises. Food production capacity should be increased, with the goal of stable yields, improved quality, and increased efficiency to ensure safe, green food production and increased income. From the perspective of policy, support policies for rice production should be improved. The market-oriented reform of food prices should be promoted, encouraging high-quality and excellent prices, broadening policy for agricultural insurance coverage and optimizing insurance operation mechanisms, as well as strengthening agricultural technology promotion, and improving the quality of extension staff. From the production perspective, scientific and technological support should be strengthened, breeding levels should be improved, the efficiency of breeding for important food traits should be enhanced, the mechanization of the food cultivation and harvesting process should be improved, and meteorological data and pest and disease data from smart agriculture should be used to help production. Rice fields should be constructed to a high standard, and water conservancy facilities should be constructed in food fields.

Local governments should actively explore the combination of fiscal instruments and finance, making full use of market mechanisms and the leverage of limited fiscal funds to leverage more social capital to participate in the production and processing of green food and extend the food-value chain. These measures could include taking the lead in the establishment of a local green food enterprise and project bank, participating in the establishment of a green food industry fund, taking the lead in setting up a green food guarantee fund, and tilting inclusive financial policies towards the green food sector. Financial institutions also need to play a greater role in innovating green financial services to support the development of the green food industry. It is recommended that financial institutions whose main business is to serve the “three rural areas”, including the Agricultural Development Bank, the Agricultural Bank of China, the Postal Savings Bank, and the local agricultural and commercial banks, take the initiative to explore standards and product innovation in terms of financial support for green food, and give full play to the synergy between green finance and inclusive finance in the process.

The government can curb the non-food cultivation trend by renting land from farmers, whereby the government leases their land from local farmers and pays them a suitable rent. This can not only increase the proportion of food grown on cropland, but also unify the use of farmers’ fragmented cropland, which facilitates the use of mechanized equipment, improves the mechanization of the agricultural production process, reduces the input of agricultural labor, and frees the farmers from farming work, so that they can devote more time to non-farm work. Farmers may also receive rent from the government and increase their income; this can be a way to kill two birds with one stone.

Furthermore, as Zhou Qiren said, the revitalization of the countryside must not focus on the countryside alone; revitalization work lies outside the countryside in the need to increase farm household income through off-farm employment. Therefore, the government should take measures to provide a more adequate supply of non-farm jobs for farming households, so that they have more opportunities to participate in non-farm work. The government should also help low-income farmers by providing them with employment guidance and relevant skills training to help them to achieve better non-farm employment. Meanwhile, specific measures should also be taken by the government to increase agricultural productivity and the level of mechanization, particularly for food crops, to generate surpluses for the large agricultural population. This would enable more people in agriculture to take up non-farm employment, thereby raising their income levels (OECD 2013).

The disadvantage of this study is that, due to the lack of data, we cannot conduct a complete intermediary exploration of the conduction paths in the “Theoretical framework” section. Additionally, conduction mechanisms can only be tested as far as is possible based on the available data. In the conduction path, after farmers increased the proportion of cash crops, the impact on the farm labor time and non-farm labor time was based on the fact that cash crops require more labor time than food crops and a summary of the previous literature [1,10]. Therefore, in future research, on the basis of the availability of data, the impact of cash crop planting on farmers’ farm labor and non-farm labor input time could be the research direction. This would help farmers to more comprehensively consider the impact of planting cash crops or food crops, and provide a reference for policy formulation.

## 7. Conclusions

This study analyzed the impact of non-food cultivation on farmers’ income, using data from the Statistical Yearbook of Zhejiang and the statistical yearbooks of the prefecture-level cities. The OLS model was used to analyze its influence.

The results estimated from the OLS model reveal that increasing the proportion of cash crop cultivation has a significantly negative impact on farmers’ total household income. Further, the disaggregated analyses showed that this non-food cultivation in Zhejiang has a more serious negative effect on lower-income farmers; the lower farmers’ income, the more severe its negative impact, while the negative effect was not significant for upper-income farmers.

Our findings provide empirical evidence for the development of cropland use policies in Zhejiang. The negative and significant impact of non-food cultivation on farmers’ household income suggests that the government should make an effort to take farmers out of the inertia of thinking that cash crop farming increases their total income and develop food-growing subsidy policies to encourage farmers to grow labor-saving crops. The lower farmers’ income, the more severely they are affected by the negative effects of non-food cultivation. This suggests that government departments should establish targeted help to low-income farmers to free them from cash crop farming. This could include training them in relevant skills and giving them as much access as possible to non-farm employment opportunities, on an equal footing with farmers of other income levels. The development of rural industries should be strengthened, and the transfer of labor-intensive industries from urban to rural areas should be promoted, so that farmers have more opportunities for employment close to home. This would help to ensure farmers’ sustainable employment and increase their income.

## Figures and Tables

**Table 1:** Variable descriptions and descriptive statistics.

Variable	Definition	Mean	Std. Dev.	Max	Min
Disposable income	Annual disposable income level of rural residents	9.3663	0.6514	10.3713	8.3248
Percentage of cash crops sown	Cash crops sown area as a percentage of total sown area	0.4789	0.0394	0.5112	0.3528
GDP	Regional overall economic development level	10.0746	0.7419	11.0762	8.7227
Mechanization level	Mechanized total power per unit sown area	0.9245	0.1830	1.2151	0.5599
Education level	Average years of education for farmers	8.3325	0.4196	9.1448	7.6676
Primary industry employment	The total number of employed persons in the primary industry	6.2658	0.4434	6.8773	5.3375
Urbanization	Urban population as a percentage of total population	0.6617	0.0619	0.7220	0.5247
Road mileage	Regional highway mileage	11.3737	0.4154	11.7206	10.6447

**Table 2:** Impact of non-food cultivation on non-farm income and farm income.

Variable	Off-Farm Income	Farm Income
Percentage of cash crops sown	−1.2752 ** (0.4394)	−0.0144 (0.2819)
GDP	0.4024 ** (0.1612)	0.4979 *** (0.0806)
Mechanization level	0.6868 *** (0.2026)	−0.2464 ** (0.1080)
Educational level	0.6915 ** (0.2470)	−0.0356 (0.1312)
Primary industry employment	−0.1033 (0.3947)	0.0703 (0.2062)
Urbanization	1.3949 (2.0315)	1.8069 (1.1104)
Road mileage	−0.2409 ** (0.0847)	0.0896 * (0.0447)
Cons	1.3794 (5.1168)	1.2390 (2.5893)
P	0.0000	0.0000
R^2^	0.9970	0.9969

Note: ***, ** and * denote statistical significance of 1%, 5%, and 10%, respectively.

**Table 3:** Impact of non-food cultivation on the household income of farm households in different income brackets.

Variable	Overall Income	Low-Income	Sub-Lower-Income	Middle-Income	Sub-High-Income	Upper-Income
Percentage of cash crops sown	−0.6393 ** (0.2743)	−1.5659 ** (0.6627)	−1.3348 * (0.6637)	−1.2572 ** (0.5409)	−0.9658 ** (0.3552)	−0.1226 (0.2026)
GDP	0.4122 *** (0.0991)	0.1015 (0.2872)	0.3304 ** (0.1475)	0.4018 *** (0.1274)	0.4693 *** (0.0986)	0.5320 *** (0.0667)
Mechanization level	0.3879 *** (0.1296)	0.6031 ** (0.2345)	0.9128 *** (0.2226)	0.8135 *** (0.2116)	0.6150 *** (0.1644)	−0.1351 (0.1568)
Education level	0.4757 *** (0.1436)	0.6148 (0.3898)	0.5561 ** (0.2366)	0.5207 ** (0.2060)	0.4694 ** (0.1667)	0.3263 *** (0.1063)
Primary industry employment	−0.1329 (0.2400)	−0.1179 (0.4665)	−0.2533 (0.4044)	−0.1012 (0.3612)	0.0132 (0.2795)	−0.5573 (0.1331)
Urbanization	1.1185 (1.4464)	1.9813 (2.3089)	−0.0084 (2.5593)	0.3817 (2.3295)	0.8830 (1.9152)	1.6636 * (0.9273)
Road mileage	−0.1475 ** (0.0575)	−0.0361 (0.1815)	−0.2115 ** (0.0881)	−0.1960 ** (0.0694)	−0.1807 *** (0.0582)	−0.0567 (0.0415)
Cons	2.9763 (3.1066)	2.0959 (6.6950)	4.6830 (5.1237)	3.2849 (4.5960)	2.2471 (3.5529)	2.1586 (1.7703)
P	0.0000	0.0000	0.0000	0.0000	0.0000	0.0000
R^2^	0.9985	0.9906	0.9948	0.9959	0.9975	0.9984

Note: ***, ** and * denote statistical significance of 1%, 5%, and 10%.

**Table 4:** Robustness test 1: Estimation of instrumental variables.

Variable	Overall Income	Low-Income	Sub-Low-Income	Middle-Income	Sub-High-Income	Upper-Income
Percentage of cash crops sown	−0.8465 *** (0.1765)	−2.0509 *** (0.4209)	−2.0230 *** (0.3664)	−1.7753 *** (0.3238)	−1.2628 *** (0.2373)	−0.1816 (0.2286)
GDP	0.4353 *** (0.0803)	0.1557 (0.2348)	0.4081 *** (0.1369)	0.4597 *** (0.1124)	0.5026 *** (0.0845)	0.5386 *** (0.0495)
Mechanization level	0.3952 *** (0.1038)	0.6202 *** (0.3070)	0.9373 *** (0.1811)	0.8317 *** (0.1690)	0.6254 *** (0.1304)	−0.1330 (0.1274)
Education level	0.4603 *** (0.1156)	0.5787 ** (0.3070)	0.5044 *** (0.1834)	0.4821 *** (0.1631)	0.4391 *** (0.1326)	0.3219 *** (0.0816)
Primary industry employment	−0.1492 (0.1989)	−0.1558 (0.3793)	−0.3077 (0.3349)	−0.1417 (0.2974)	−0.0100 (0.2301)	−0.0619 (0.1118)
Urbanization	0.9631 (1.2569)	1.6177 (2.0422)	−0.5296 (2.2575)	−0.0067 (2.0241)	0.6604 (1.6437)	1.6193 ** (0.7939)
Road mileage	−0.1514 *** (0.0495)	−0.0452 (0.1517)	−0.2245 *** (0.0805)	−0.2057 *** (0.0623)	−0.1863 *** (0.0509)	−0.0578 * (0.0457)
Cons	3.2064 (2.5693)	2.6345 (5.4040)	5.4551 (4.1907)	3.8603 (3.7459)	2.5768 (2.9082)	2.2241 * (1.4950)
P	0.0000	0.0000	0.0000	0.0000	0.0000	0.0000
R^2^	0.9984	0.9904	0.9945	0.9957	0.9974	0.9984

Note: ***, ** and * denote statistical significance of 1%, 5%, and 10%, respectively.

**Table 5:** Robustness test 2: Change in explanatory variables.

Variable	Overall Income	Low-Income	Sub-Low-Income	Middle-Income	Sub-High-Income	Upper-Income
Percentage of cash crops sown	−0.8568 ** (0.3892)	−1.0284 ** (0.3998)	−1.0633 ** (0.4109)	−1.2441 ** (0.5154)	−0.5008 ** (0.2735)	−0.1333 (0.2687)
GDP	0.3769 *** (0.0609)	0.1994 ** (0.0854)	0.1912 ** (0.0846)	0.3390 * (0.1550)	0.5219 *** (0.0785)	0.5003 *** (0.0436)
Mechanization level	0.2353 * (0.1132)	0.2847 ** (0.1262)	0.2393 ** (0.1116)	0.5880 ** (0.2240)	0.1128 (0.0963)	0.0061 (0.0716)
Education level	0.4393 *** (0.1231)	0.2175 (0.2683)	0.2044 (0.1427)	0.5586 * (0.2894)	0.3856 ** (0.1398)	0.3043 *** (0.0938)
Primary industry employment	−0.1959 (0.1831)	−0.8680 *** (0.2682)	−1.0721 *** (0.2235)	−0.2778 (0.4052)	−0.1176 (0.1750)	−0.1180 (0.1196)
Urbanization	1.1486 (1.5671)	0.4822 (1.6330)	−0.0750 (1.4923)	0.3519 (2.8311)	0.0828 (1.4269)	1.3122 (0.8681)
Road mileage	−0.0433 (0.0410)	−0.0681 (0.0658)	−0.0443 (0.0599)	−0.2022 ** (0.0891)	−0.1173 ** (0.0470)	−0.0324 (0.0261)
Cons	3.1729 (2.3131)	10.9755 *** (3.0955)	13.0573 *** (2.7118)	5.1896 (5.1860)	3.3224 (2.1480)	3.0779 * (1.5531)
P	0.0000	0.0000	0.0000	0.0000	0.0000	0.0000
R^2^	0.9984	0.9972	0.9984	0.9948	0.9983	0.9995

Note: ***, ** and * denote statistical significance of 1%, 5%, and 10%, respectively.

**Table 6:** Robustness test 3: Additional variables method.

Variable	Overall Income	Low-Income	Sub-Low-Income	Middle-Income	Sub-High-Income	Upper-Income
Percentage of cash crops sown	−0.7462 ** (0.3218)	−1.9458 ** (0.8642)	−1.4733 * (0.7520)	−1.3713 ** (0.6184)	−1.1013 ** (0.4096)	−0.1150 (0.2759)
GDP	0.4302 *** (0.0960)	0.0947 (0.2529)	0.3654 * (0.1793)	0.4282 ** (0.1523)	0.5060 *** (0.1219)	0.4985 *** (0.0873)
Mechanization level	0.3512 (0.2332)	0.7167 (0.4784)	0.8248 (0.4701)	0.7492 (0.4255)	0.5206 (0.3116)	−0.0210 (0.2264)
Education level	0.5211 *** (0.1659)	0.7272 (0.4110)	0.6231 * (0.3119)	0.5742 * (0.2672)	0.5283 ** (0.2237)	0.3008 ** (0.1262)
Primary industry employment	−0.0999 (0.2012)	−0.1465 (0.4705)	−0.1863 (0.4627)	−0.0510 (0.3806)	0.0837 (0.2615)	−0.1264 (0.1474)
Urbanization	0.8326 (1.9393)	1.4233 (3.4349)	−0.4548 (3.1425)	0.0294 (2.9908)	0.4310 (2.5749)	1.8931 (1.3538)
Road mileage	−0.1518 * (0.0738)	−0.0978 (0.1796)	−0.2095 (0.1197)	−0.1959 * (0.1059)	−0.1772 * (0.0826)	−0.0775 (0.0448)
Sex	1.5501 (1.8686)	6.1389 (6.6838)	1.9057 (3.3481)	1.5921 (2.9061)	1.8413 (2.2301)	0.1766 (1.6408)
Household size	−0.0086 (0.1605)	0.1124 (0.2529)	−0.0347 (0.2530)	−0.0237 (0.2424)	−0.0388 (0.1919)	0.0658 (0.0891)
Cons	1.8185 (3.1334)	−0.7290 (7.9401)	2.9686 (6.8190)	1.9158 (5.6535)	0.5273 (4.0124)	2.8301 * (2.4115)
P	0.0000	0.0000	0.0000	0.0000	0.0000	0.0000
R^2^	0.9985	0.9918	0.9949	0.9959	0.9975	0.9985

Note: ***, ** and * denote statistical significance of 1%, 5%, and 10%, respectively.

## Data Availability

Not applicable.

## References

[1] Li M., Gan C., Ma W.L., Jiang W. (2020). Impact of cash crop cultivation on household income and migration decisions: Evidence from low-income regions in China. J. Integr. Agric..

[2] Poulton C., Al-Hassan R., Cadish G., Reddy C., Smith L. (2001). The Cash Crop Versus Food Crop Debate, Issues Paper-3, Crop Post-Harvest Programme.

[3] Ruerd R., Marrit V.D.B. (2001). Nonfarm employment and poverty alleviation of rural farm households in Honduras. World Dev..

[4] Masanjala W.H. (2006). Cash crop liberalization and poverty alleviation in Africa: Evidence from Malawi. Agric. Econ..

[5] Radchenko N., Corral P., Winters P. (2018). Heterogeneity of commercialization gains in the rural economy. Agric. Econ..

[6] Arndt C., Benfica R., Tarp F., Thurlow J., Uaiene R. (2010). Biofuels, poverty, and growth: A computable general equilibrium analysis of Mozambique. Environ. Dev. Econ..

[7] Klasen S., Priebe J., Rudolf R. (2013). Cash crop and income dynamics in rural areas for post-crisis Indonesia. Agric. Econ..

[8] Von B.J. (1995). Agricultural commercialization: Impact on income and nutrition and implications for policy. Food Policy.

[9] Kennedy E., Peters P. (1992). Household food security and child nutrition: The interaction of income and gender of household head. World Dev..

[10] Tankari M.R. (2017). Cash crops reduce the welfare of farm households in Senegal. Food Secur..

[11] Anderman T.L., Remans R., Wood S.A., DeRosa K., DeFries R.S. (2014). Synergies and tradeoffs between cash crops production and food security: A case study in rural Ghana. Food Secur..

[12] Achterbosch T.J., Van Berkum S., Meijerink G.W. (2014). Cash Crops and Food Security: Contributions to Income, Livelihood Risk and Agricultural Innovation.

[13] Ali A., Rahut D.B. (2018). Farmers willingness to grow GM food and cash crops: Empirical evidence from Pakistan. GM Crops Food-Biotechnol. Agric. Food Chain.

[14] Peng J.Q., Zhao Z.H., Liu D.N. (2022). Impact of agricultural mechanization on agricultural production, income, and mechanism: Evidence from Hubei province, China. Front. Environ. Sci..

[15] Hussain A., Memom J., Alam H.S. (2020). Weather shock, coping strategies and farmers’ income: A vase of rural areas of district Multan, Punjab. Weather. Clim. Extrem..

[16] Nahar A., Luckstead J., Wailes E.J., Alam M.J. (2018). An assessment of the potential impact of climate change on rice farmers and markets in Bangladesh. Clim. Chang..

[17] Wu W.H., Wu G.S., Yin C.B., Chien H.P. (2020). Impact of farming on farmer’s income: A case of Wuchang rice in China. Jpn. Agric. Res. Q..

[18] Kahane R., Hodgkin T., Jaenicke H., Hoogendoorn C., Herman M., d’Arros Hughes J., Padulosi S., Looney N. (2013). Agrobiodiversity for food security, health and income. Agron. Sustain. Dev..

[19] Porter G., Howard K.P. (1997). Comparing contract: An evaluation of contract farming schemes in Africa. World Dev..

[20] Ellis F. (1988). Peasant Economics: Farm Households and Agrarian Development.

[21] Asfaw S., Shiferaw B., Simtowe F., Lipper L. (2012). Impact of modern agricultural technologies on smallholder welfare: Evidence from Tanzania and Ethiopia. Food Policy.

[22] Cuong N.V. (2009). Measuring the impact of cash crops on household expenditure and poverty in rural Vietnam. Asia Pac. Dev. J..

[23] Ojo T.O., Baiyegunhi L.J.S. (2021). Climate change perception and its impact on net farm income of smallholder rice farmers in South-west, Nigeria. J. Clean. Prod..

[24] Osewe M., Liu A.J., Njagi T. (2020). Farmers-led irrigation and its impacts on smallholder farmers’ crop income: Evidence from southern Tanzania. Int. J. Environ. Res. Public Health.

[25] Brück T. (2004). The welfare effects of farm household activity choices in Post-War Mozambique. HICN Working Paper No. 04.

[26] Grootaert C. (1997). Determinants of poverty in Côte D’Ivoire in the 1980s. J. Afr. Econ..

